# Ultrasonic-Formic Acid Pretreatment Coupled with Metal Ion/Deep Eutectic Synergistic Catalysis: Efficient Conversion of Biomass to 5-Hydroxymethylfurfural

**DOI:** 10.3390/polym18020218

**Published:** 2026-01-14

**Authors:** Xiaowei Zhuang, Yue Liu, Zhijun Wu, Yongshun Feng, Xin Pan, Hui Qiao

**Affiliations:** 1 Zhejiang Academy of Forestry, Liuhe Road 399, Hangzhou 310023, China; zhuangxiaowei@zjforestry.ac.cn (X.Z.); 472321897@stu.lntu.edu.cn (Y.L.); fengyongshun@zjforestry.ac.cn (Y.F.); panxin@zjforestry.ac.cn (X.P.); 2 College of Environmental and Engineering, Liaoning Technical University, Zhonghua Road 47, Fuxin 123008, China; wuzhijun@lntu.edu.cn

**Keywords:** biomass, ultrasonic-formic acid pretreatment, 5-hydroxymethylfurfural, deep eutectic solvent, Lewis acid

## Abstract

This study developed a two-step conversion strategy for the efficient conversion of bamboo waste into 5-hydroxymethylfurfural (HMF). First, ultrasonic-assisted formic acid pretreatment was used at 80 °C for 3 h, removing approximately 83.7% of hemicellulose and 76.5% of lignin from the biomass, with a cellulose recovery of 93.5%. The ultrasonic step significantly enhanced the chemical action of formic acid through cavitation, allowing formic acid to penetrate deeper into the biomass, thereby more effectively removing hemicellulose and lignin. Subsequently, glucose was obtained through an enzymatic hydrolysis. In the second step of HMF preparation, citric acid in the hydrolysate was combined with ChCl to form an acidic deep eutectic solvent (DES), and metal chlorides were added as Lewis acid catalysts. Experiments results showed that when the ChCl–citric acid ratio was 2:1, and the Ca^2+^ concentration was 100 mM, an HMF yield of 51.9% was obtained at 220 °C for 1.5 h. This study provides an efficient, mild, and environmentally friendly method for the high-value valorization of waste bamboo.

## 1. Introduction

As the distribution center of bamboo in the world, China possesses a significant resource advantage, with a bamboo forest area reaching 70.197 million hm^2^ [1]. Bamboo, as a representative lignocellulosic biomass, constitutes an abundant renewable resource. However, its utilization rate remains low due to the substantial quantities of harvesting and processing residues during industrial operations. The chemical conversion of bamboo into high-value products is of considerable significance, as lignocellulosic biomass, one of the most abundant natural carbon source and a sustainable energy reservoir, can be transformed into liquid fuels and a variety of value-added compounds [2,3].

5-Hydroxymethylfurfural (HMF) is a key biomass-derived platform compound whose true industrial leverage lies its ability to deliver short, high-yield routes to polymer-grade furan monomers. The most direct t example is the quantitative oxidation of HMF to 2,5-furandicarboxylic acid (FDCA) in replacement for terephthalic acid, whose polycondensation with ethylene glycol yields polyethylene furanoate (PEF), a next-generation polyester exhibiting gas-barrier performance and higher glass-transition temperature [4,5]. Beyond FDCA, HMF is readily converted into 2,5-diformylfuran (DFF) and 2,5-bis(hydroxymethyl)furan for the synthesis of high-Tg furanic epoxies and polyurethanes [6,7]. Collectively, these furan-based intermediates offer a direct, renewable gateway to industrial polycondensation and polyaddition processes, allowing fossil-derived carbon feedstocks to be seamlessly substituted in large-volume polymer applications [8]. Nevertheless, achieving large-scale commercial replacement of petroleum-based products requires more efficient biomass conversion technologies. Lim et al. reported a one-step synthesis of HMF from bamboo using microwave-assisted H_2_SO_4_ heating, yielding 10.94% HMF at 140 °C for 5 min [9]. Hoşgün et al. established a one-step hydrothermal process for converting poppy straw to HMF with metal chloride catalysts, achieving a yield of 12.24% using CuCl_2_ (0.03 M) at 200 °C for 30 min [10]. Due to the generally low efficiency of one-step methods, biomass conversion is often separated into two optimized phases to improve overall yield. Hoang et al. demonstrated an ultrasound-assisted strategy employing HSO_3_-ZSM-5 zeolite after NaOH pretreatment, yielding 54.1% HMF from rice straw. Sweygers et al. obtained 37% and 35% HMF from bamboo culm and leaves, respectively, applying 0.13 M HCl and 177 °C under microwave heating [11,12]. The comparison with this work was shown in Table 1.

In summary, biomass represents a low-cost feedstock for HMF production, and enzymatic hydrolysis provides a mild, efficient, and environmentally benign conversion pathway. However, lignocellulosic biomass is composed of structural polymers interconnected by rigid and recalcitrant chemical bonds, making them highly resistant to enzymatic or chemical attack [13]. Consequently, the development of efficient and economically viable pretreatment strategies capable of disrupting this complex architecture remains a major challenge for the high-value utilization of biomass feedstocks. Traditional chemical pretreatments, such as dilute-acid and alkaline hydrolysis, exhibit strong deconstruction capabilities but also pose drawbacks, including high corrosiveness, environmental hazards, equipment degradation, and difficult downstream separation [14]. Formic acid, a biodegradable organic acid, can efficiently remove lignin and hemicellulose under mild conditions and can be readily recovered via vacuum distillation, making it a promising low-cost and environmentally friendly pretreatment reagent. Ultrasound-assisted pretreatment, driven by acoustic cavitation, further enhances lignocellulosic disruption, increasing cellulose accessibility and reactivity. Previous studies have demonstrated that this approach significantly improves enzymatic hydrolysis efficiency while lowering reaction temperatures and processing times. Owing to its simplicity, low energy requirements, and environmental benignity, ultrasound-assisted pretreatment offers a sustainable and effective route for biomass pretreatment [15,16].

Efficient production of HMF requires both suitable pretreatment and effective catalytic systems. The conversion of glucose to HMF proceeds through two essential steps: Lewis acid–catalyzed isomerization of glucose to fructose, followed by Brønsted acid–catalyzed dehydration of fructose to HMF [17]. However, designing catalytic systems that simultaneously provide both Lewis and Brønsted acidity remains a significant challenge. Currently, most HMF production strategies rely on metal chlorides as Lewis acid catalysts, either alone or in combination with mineral Brønsted acids such as sulfuric acid or hydrochloric acid, to facilitate carbohydrate dehydration [18].

Recently, deep eutectic solvents (DESs) have attracted considerable interest due to their low toxicity, biodegradability, simple synthesis, cost effectiveness, and non-flammability [19]. DESs typically consist of a hydrogen-bond acceptor (HBA) and a hydrogen-bond donor (HBD), and they exhibit physical and chemical properties similar to ionic liquids. Among the various classes of DESs, Type III DESs—comprising organic salts as HBAs paired with HBDs—have received the most attention [20]. Previous studies have demonstrated that HMF possesses high solubility and excellent stability in DESs, making DES-based catalytic environments highly advantageous for HMF production [21].

In this study, a highly effective two-step treatment strategy was developed for the production of HMF. To obtain solid residues with enhanced enzymatic accessibility, bamboo was first pretreated using an ultrasound–formic acid process to disrupt and loosen its dense lignocellulosic structure. Subsequently, citric acid present in the enzymatic hydrolysate served as a hydrogen-bond donor to form a deep eutectic solvent (DES) with choline chloride, the hydrogen-bond acceptor, enabling catalytic conversion of glucose into HMF. FT-IR, XRD, SEM, and XPS analyses were conducted to elucidate the effects of ultrasound–formic acid pretreatment on the physicochemical structure of bamboo. Furthermore, a mechanistic pathway was proposed for the DES-assisted, metal-chloride-catalyzed transformation of enzymatic hydrolysates within the ChCl–citric acid system. Overall, this work provides an efficient ultrasound–formic acid pretreatment strategy and demonstrates its potential for converting cellulose-rich biomass into HMF.

## 2. Materials and Methods

### 2.1. Materials

Bamboo was harvested from Anji, Zhejiang, China. The bamboo was dried at 105 °C for 6 h and crushed below 40 mesh before use. Anhydrous formic acid, sodium carbonate, ChCl, CuCl_2_, AlCl_3_, FeCl_3_, BaCl_2_, NaCl, KCl, MnCl_2_, MgCl_2_ and CaCl_2_ were purchased from Chengdu Kelong Chemical Co., Ltd. (Chengdu, China) and Macklin Reagent Co., Ltd. (Shanghai, China).

### 2.2. Ultrasonic-Formic Acid Pretreatment

The dried bamboo powder (5 g) was mixed with 50 mL of 90% (*v*/*v*) formic acid and placed in a 250 mL Erlenmeyer flask (Synthware, Chongqing, China). The mixture was ultrasonic for 1 h and then heated at different temperatures for various time at a speed of 120 rpm using a water bath. The ultrasonic treated bamboo powder was named US-BP, and the ultrasonic-formic acid treated bamboo powder was named US-FA-BP. After pretreatment, the mixture was separated by a Buchner funnel, and the solid residue was washed with formic acid to remove residual sugar and lignin from the surface and then washed with water until neutral. Before enzymatic hydrolysis, the pretreated bamboo powder was mixed with 50 g/L sodium carbonate solution at a ratio of 1:10, soaked for 20 min, and the solid residue was filtered after soaking, washed with distilled water until neutral according to previous study [3].

### 2.3. Enzymatic Hydrolysis

The enzymatic hydrolysis was performed in sodium citrate buffer (pH 4.8) with a solid loading of 5% substrate (*w*/*v*) using a 100 mL dense Erlenmeyer flask, and 20 FPU/g glucan cellulase (Cellic Ctec2, Novozyme, Beijing, China) was added. Then, the mixture was enzymatically hydrolyzed at 50 °C and 150 rpm for 72 h. All digestion experiments were performed in duplicate.

### 2.4. The Preparation of HMF from Enzymatic Hydrolysate

The enzymatically hydrolyzed solid–liquid mixture was separated using a centrifuge at 8000 rpm for 15 min, and the supernatant was collected for later use. A muffle furnace (L5/11/B180, Naberthem GmbH, Lilienthal, Germany) was used to produce HMF from the enzymatic hydrolysate at high temperatures in a 5 mL stainless steel reactor lined with Teflon.

1.8 mL of the enzymatic hydrolysate and 0.2 mL of 1 mol/L different metal chloride ions and an appropriate amount of ChCl were mixed, and the reactor was heated to the target temperature, and the specified reaction time was maintained. After the reaction, the reactor was naturally cooled to room temperature.

### 2.5. Methods of Analysis

#### 2.5.1. Structural Analysis of Pretreated Samples

X-ray photoelectron spectroscopy (XPS) analysis of C and O atoms on the surface of solid samples using Nexsa G2, Thermo Fisher Scientific (Waltham, MA, USA). FTIR spectra in the range of 4000–400 cm^−1^ were collected on a Bruker Vertex 80 v spectrophotometer (Bruker, Ettlingen, Germany) at room temperature. The surface topography of the samples was determined using the S-3400N, Hitachi Limited Scanning Electron Microscope (SEM) (Tokyo, Japan). The crystallinity analysis of the samples was performed using an XRD-6100 ray diffractometer (Shimadzu, Kyoto, Japan) with an X-ray diffraction (XRD) obtained from 5° to 40° at a rate of 5°/min, and the crystallinity index (CrI) was obtained according to the previous study [22].

(1) $$CrI=\frac{I_{002}-I_{am}}{I_{002}}\times 100$$

where $I_{002}$ denotes the diffraction intensity of the crystalline region (2θ = 22.5°) of the cellulose component in the biomass, $I_{am}$ denotes the diffraction intensity of the amorphous region (2θ = 18.5°) of the cellulose component in the biomass.

#### 2.5.2. Other Methods of Analysis

The composition of untreated and treated samples was determined according to the National Renewable Energy Laboratory standard analytical method [23]. Glucose and HMF concentrations were quantified by high-performance liquid chromatography (HPLC, Agilent 1260, Santa Clara, CA, USA) using an Aminex Bio-Rad HPX-87H (Hercules, CA, USA) column and a refractive index detector. 5 mM H_2_SO_4_ was used as the mobile phase, the flow rate was controlled at 0.6 mL/min at 55 °C, and the refractive index detector detected the analysis signal. The conversion yield of glucose to HMF is calculated as follows.

(2) Yield of HMFmol%=mHMF126mglucose180×100

The $m_{HMF}$ represents the concentration of glucose in the liquid before the reaction, and $m_{glucose}$ represents the concentration of HMF in the liquid after the reaction, 126 and 180 are the molar mass of HMF and glucose, respectively.

## 3. Results and Discussion

### 3.1. Effect of Ultrasonic-Assisted Formic Acid Pretreatment on Bamboo Powder

**Figure 1:**
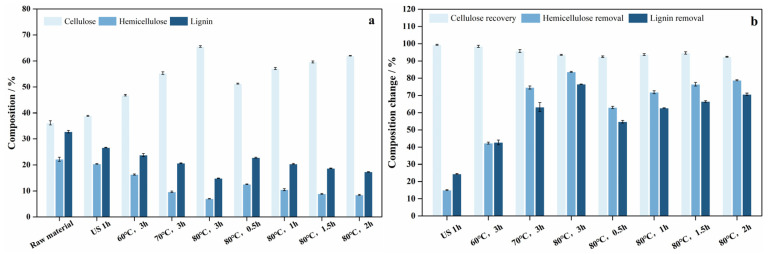
Effects of different pretreatment conditions on the composition of pretreatment bamboo powder. (**a**) Composition of raw material and pretreated bamboo powder, (**b**) Composition changes of raw material and pretreated bamboo powder.

To investigate the impact of formic acid pretreatment on the chemical composition of bamboo powder, 90% (*v*/*v*) formic acid was applied at an L/S ratio of 10:1 following 1 h of ultrasonic treatment. The effect of formic acid pretreatment under different conditions were subsequently compared. The changes in the chemical composition of bamboo powder after pretreatment are shown in Figure 1. The untreated bamboo powder consisted of 36.2% cellulose, 22.2% hemicellulose, and 32.8% lignin. After 1 h of ultrasonic treatment, the composition changed to 38.9% cellulose, 20.4% hemicellulose and 26.7% lignin, corresponding to the dissolution of 15.02% hemicellulose and 24.42% lignin, indicating that ultrasonication facilitates the penetration of formic acid into the dense bamboo structure and partially disrupts its chemical matrix. Subsequently, as the temperature increased and the pretreatment duration was extended, the hemicellulose and lignin contents decreased significantly, falling below 7% and 15% at 80 °C for 3 h, respectively. Simultaneously, the proportion of cellulose gradually increased, reaching 65.5%. From Figure 1b, more than 92% cellulose was retained, while most hemicellulose and lignin were removed by formic acid, except under the condition of 60 °C for 3 h. Under the optimal condition of 80 °C for 3 h, the removal rates of hemicellulose and lignin reached 83.7% and 76.5%, respectively, with a cellulose recovery of 93.5%. These results indicate that the initial ultrasonic treatment facilitates subsequent formic acid–induced cleavage of the bamboo chemical structure, enabling efficient component separation under mild conditions.

### 3.2. Enzymatic Hydrolysis of Ultrasonic-Formic Acid Pretreated Bamboo Powder

**Figure 2:**
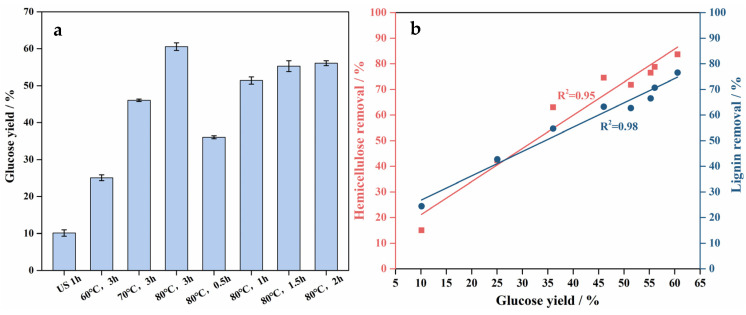
(**a**) Glucose yield after enzymatic hydrolysis under different treatment conditions. (**b**) The relationship between hemicellulose and lignin removal rate and glucose yield.

Figure 1 shows that hemicellulose and lignin were progressively removed with increasing temperature and time, thereby improving cellulose accessibility. Previous studies have demonstrated that increased cellulose accessibility significantly enhances its enzymatic hydrolysis [24]. Based on this, the effects of formic acid pretreatment under different temperatures and durations on the enzymatic hydrolysis of bamboo powder were investigated, and the results are presented in Figure 2. The glucose yield increased with rising temperature and extended pretreatment time, with temperature exhibiting a more pronounced influence. The highest glucose yield of 60.6% was achieved at 80 °C for 3 h. In general, both lignin and hemicellulose are well-recognized barriers limiting cellulose hydrolysis [25]. After formic acid pretreatment, the high cellulose retention and substantial lignin removal collectively promoted the enzymatic hydrolysis of cellulose. According to previous reports, the removal of hemicellulose and lignin can substantially increase the specific surface area and pore volume of biomass, thereby improving cellulose accessibility to enzymes [26]. Figure 2b illustrates the correlation between glucose yield and the removal rates of hemicellulose and lignin, both of which exhibited strong linear relationships (R^2^ ≥ 0.95). These strong linear correlations indicate that hemicellulose and lignin removal induced by ultrasonic–formic acid pretreatment significantly facilitates cellulose enzymatic hydrolysis.

### 3.3. Physicochemical Characterization of Bamboo Powder After Pretreatment

**Figure 3:**
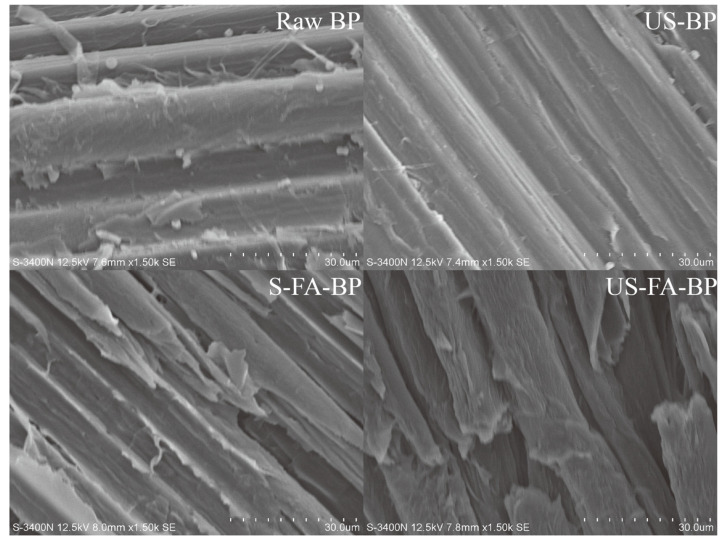
SEM microstructure images of raw materials and bamboo powder pretreated with formic acid.

The effects of ultrasound and formic acid pretreatment on the morphology of biomass were examined using SEM, as shown in Figure 3. Both the raw bamboo and the ultrasonic-treated bamboo exhibited intact and highly ordered structures. However, the bamboo treated with ultrasound for 1 h appeared smoother with slight surface ruptures compared with the raw sample, indicating that ultrasonication facilitated the partial dissolution of surface structures by formic acid. After formic acid treatment at 80 °C for 3 h, the bamboo exhibited severe structural disruption. In addition, a control experiment was conducted in which bamboo was soaked in formic acid for 1 h and subsequently heated at 80 °C for 3 h, and this sample was designated S-FA-BP. The resulting bamboo powder contained 64.6% cellulose, 7.0% hemicellulose, and 16.4% lignin, which was comparable to the composition obtained after ultrasonic–formic acid pretreatment. The surface of the soak–formic acid–treated bamboo appeared rougher with visible cracks, although no complete fiber fracture was observed. These observations suggest that ultrasonication provides a more effective pathway for formic acid to penetrate bamboo and disrupt its dense structure.

**Figure 4:**
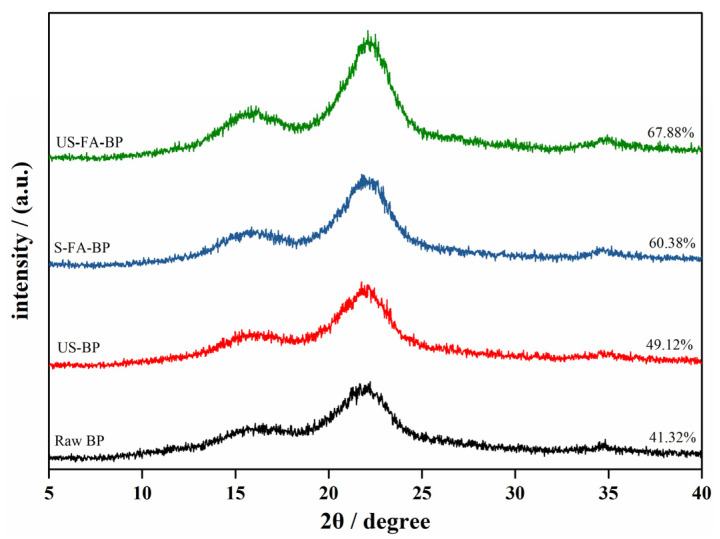
X-ray diffraction pattern of a solid residue obtained from different treatment.

The cellulose crystallinity of the four samples was analyzed, and the results are presented in Figure 4. All samples exhibited a typical cellulose I structure, with characteristic diffraction peaks at 2θ = 14.8°, 16.3°, and 22.7° [27]. The cellulose in all samples retained the cellulose I crystalline form before and after treatment; however, the proportion of the amorphous region changed. Cellulose chains are connected through hydrogen bonding to form fibrillar aggregates, and the processed samples exhibited sharper diffraction peaks at 2θ = 22.5° (I_002_) and 35° (I_40_), indicating disruption of the amorphous regions and partial hydrogen-bond cleavage following formic acid pretreatment of lignocellulosic biomass [28]. After ultrasonic treatment, the CrI increased from 41.32% in raw BP to 49.12%, likely due to enhanced formic acid penetration that disrupted amorphous cellulose and removed small amounts of hemicellulose and lignin. With the continued removal of hemicellulose and lignin, the CrI further increased to 60.38% (S-FA-BP) and 67.88% (US-FA-BP). These results indicate that formic acid pretreatment effectively removes amorphous cellulose, hemicellulose, and lignin, consistent with the substantial increase in cellulose content observed in the pretreated samples compared with the raw material.

**Figure 5:**
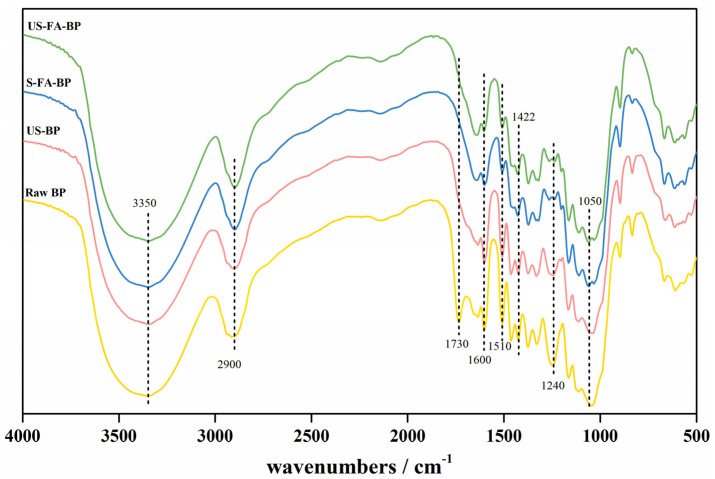
FTIR analysis of solid residues obtained by US-formic acid pretreatment.

To investigate the changes in functional groups after formic acid pretreatment the infrared spectra of the four samples were analyzed, as shown in Figure 5 and Table 2. The broad peak at about 3350 cm^−1^ corresponded to O–H stretching vibrations, while the absorption band at 2900 cm^−1^ was attributed to C–H stretching in methyl groups. The peak at 1730 cm^−1^, attributed to the C=O stretching vibration of acetyl groups, was evident in raw BP but diminished after 1 h of ultrasonication, indicating cleavage of acetyl functionalities. The bands at 1600, 1510 and 1422 cm^−1^ correspond to aromatic skeletal vibrations, and their attenuation in S-FA-BP and US-FA-BP indicates lignin removal during formic acid treatment [29,30]. The expansion vibration in 1240 cm^−1^, assigned to C–O stretching in ether linkages, was also markedly weakened due to the substantial lignin removal. The C–O stretching vibration of cellulose at 1050 cm^−1^ appeared only in S-FA-BP and US-FA-BP, demonstrating that formic acid pretreatment effectively cleaves lignin-cellulose linkages [31,32].

**Figure 6:**
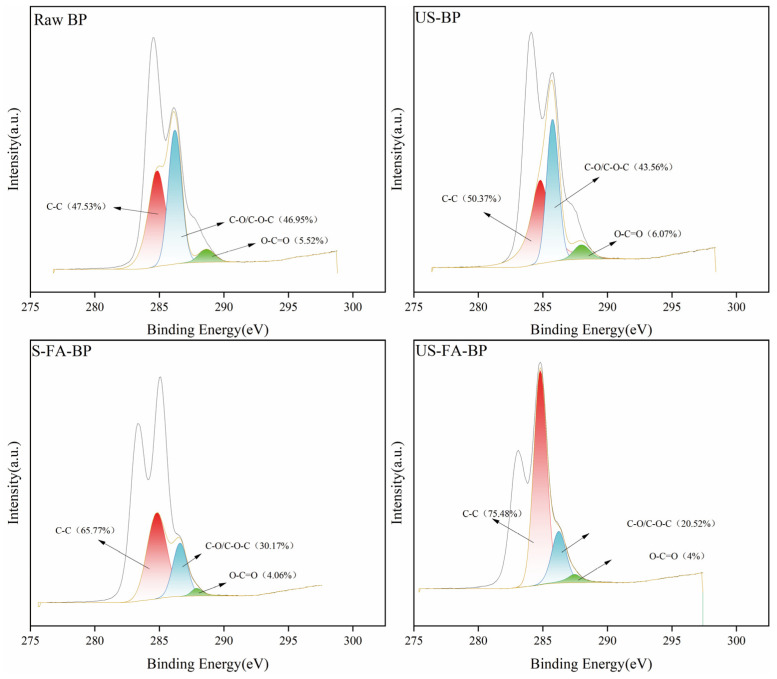
XPS analysis of solid residues obtained after pretreatment.

XPS analysis provides information on the elemental composition and bond distribution in the near-surface region of the sample. Figure 6 and Table 3 show the fitted C1s XPS spectra, which contain three characteristic components: C1, C2, and C3. The C1 component is commonly attributed to lignin-derived carbon, whereas C2 and C3 are associated with polysaccharides [30]. Both ultrasonic treatment and formic acid pretreatment promoted lignin decomposition, as indicated by an increase in oxygen content and a corresponding decrease in C1%. In US-FA-BP, the C1% value decreased markedly, indicating that this combined treatment was particularly effective in lignin degradation. Changes in C2% and C3% indicate alterations in the polysaccharide structure during pretreatment. After ultrasonication for 1 h followed by formic acid treatment at 80 °C for 3 h, the C2% value was the highest. Although C2 originates from multiple bamboo components, it is primarily associated with cellulose; thus, the increase in C2% suggests a substantially improved cellulose retention rate after pretreatment [33]. The variations in the O/C ratio and C1–C3 contents are summarized in Table 3. As pretreatment severity increased, lignin removal improved, reflected by a continuous rise in the O/C ratio and a decrease in C1%, consistent with the trends observed in the chemical composition analysis.

### 3.4. Production of HMF from Enzymatic Hydrolysate

#### 3.4.1. Effect of DES on the Production of HMF

**Figure 7:**
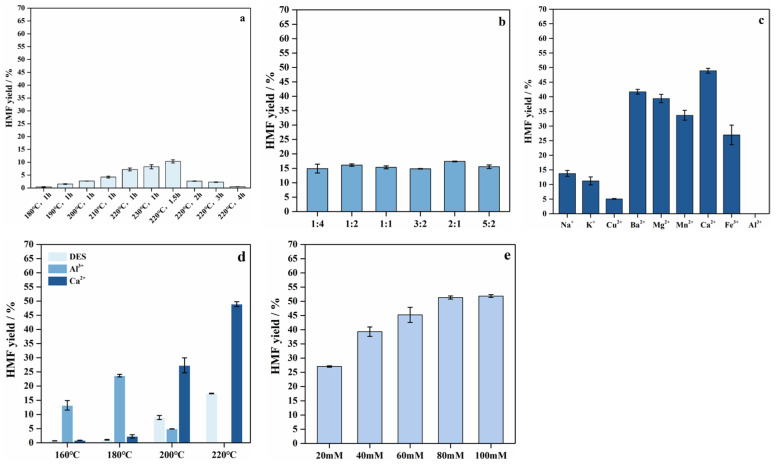
The yield of HMF prepared from enzymatic hydrolysate. (**a**) the enzymatic hydrolysate was heated directly under different temperature and time, (**b**) the effect of the ratio of citric acid to ChCl under 220 °C for 1.5 h, (**c**) the effect of different metal chloride ions under 220 °C for 1.5 h, (**d**) the effect of DES (2:1 citric acid/ChCl), Al^3+^ (100 mM) and Ca^2+^ (100 mM) under different temperature, (**e**) the effect of DES synergistic with different concentration of Ca^2+^ under 220 °C for 1.5 h.

The effects of different temperatures and reaction times on HMF yield were experimentally investigated, and the results are shown in Figure 7. The HMF yield gradually increased with rising temperature and prolonged reaction time. At 220 °C for 1.5 h, the HMF yield reached a maximum of 10.3%, whereas milder conditions (lower temperatures and shorter times) resulted in lower yields due to the accumulation of dehydration intermediates [34]. Further extension of reaction time not only promotes the conversion of glucose to HMF but also accelerates the polycondensation of HMF into polymers or other undesired by-products. Figure 7a shows that the yield of HMF increased with increasing temperature. However, excessively high temperatures can induce further polymerization of HMF and its intermediates into larger molecular fragments, thereby reducing yield. This indicates that high temperature combined with an appropriate reaction time is crucial for maximizing HMF formation [35]. On this basis, the effects of different ratios of choline chloride (ChCl) and citric acid on HMF yield were further examined, as shown in Figure 7b. When the ChCl–citric acid ratio was 2:1, the HMF yield reached its highest value of 17.4%. This result suggests that the synergistic interaction between ChCl and citric acid plays a crucial role in optimizing the HMF production process.

#### 3.4.2. Effect of Lewis Acid and DES on the Production of HMF

To further improve HMF yield, the addition of metal ions to the enzymatic hydrolysate was considered to function as Lewis acid co-catalysts for glucose conversion. Figure 7c illustrates the effects of different metal ions added to the enzymatic hydrolysate on HMF production. The results show that Ca^2+^ exhibited the highest catalytic performance yielding 48.9% HMF at 220 °C for 1.5 h, whereas no detectable HMF was produced in the presence of Al^3+^. Based on these findings, the effects of Ca^2+^ and Al^3+^ addition at different catalytic temperatures were further investigated. The addition of Al^3+^ resulted in a maximum HMF yield of 23.7% at 180 °C, whereas Ca^2+^ achieved its highest yield at 220 °C, which was substantially higher than that obtained with Al^3+^. This difference may be attributed to the tendency of Al^3+^ to promote excessive glucose degradation or side-product formation under high-temperature catalytic conditions, as Gagne and Hawthorne [36] reported that the Lewis strength of Al^3+^ (0.58) is higher than that of Ca^2+^ (0.26). In contrast, Ca^2+^ effectively catalyzes HMF formation over a broader temperature range, demonstrating strong catalytic potential [37]. García-Sancho et al. reported that the addition of CaCl_2_ significantly enhanced glucose conversion and HMF yield [38]. Ca^2+^ ions promote the formation of α-D-glucose by shifting the isomeric equilibrium, thereby accelerating glucose conversion and HMF production. In this catalytic system, Ca^2+^ not only promotes glucose isomerization but also significantly improves the selectivity and yield of HMF.

Finally, DES and Ca^2+^ were selected as synergistic catalysts for glucose conversion to HMF, and different concentrations of Ca^2+^ were added in the enzymatic hydrolysate. The results showed that increasing Ca^2+^ concentration led to a continuous rise in HMF yield, from 39.3% to 51.9%. When the Ca^2+^ concentration reached 80 mM, the HMF yield approached a plateau, reaching 51.3%. In general, the catalyst loading exerts a strong influence on the yield of the target product. Notably, when the catalyst dosage increases to a certain threshold, the HMF yield improves markedly and reaches a maximum at an optimal concentration. However, further increases in catalyst dosage resulted in no additional improvement or even a reduction in yield. One possible explanation is that excess catalyst lowers the activation energy required for the reaction, altering the reaction pathways and promoting side reactions [39]. Therefore, optimal HMF production is achieved at a specific Ca^2+^ concentration. Overall, Ca^2+^ demonstrates excellent catalytic performance as a Lewis acid, and its synergistic interaction with the DES system further enhances HMF yield.

HMF was isolated from the reaction mixture by extraction with dichloromethane or ethyl acetate, and the extraction rates were 90.19% and 85.92%, respectively, affording monomer-grade purity suitable for direct polymerization. Righetti et al. [21] previously demonstrated that HMF remains stable and highly soluble in the similar DES family used here, confirming that the extracted product can be fed into downstream FDCA/PEF or furanic-resin syntheses without additional stabilization steps.

### 3.5. Proposed Mechanism of HMF Formation from Bamboo Powder by Pretreatment and Lewis Acid Synergistic Effect

**Figure 8:**
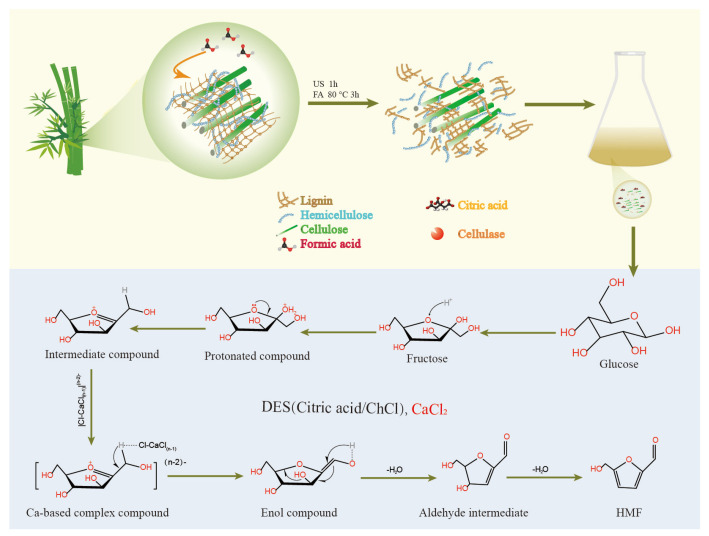
Mechanism diagram of formic acid pretreatment and high-temperature catalytic preparation of HMF from bamboo.

It has been reported that metal salt dissolved in water coordinate with water molecules to form hydrated metal–ligand complexes, [M(H_2_O)_n_]^z^, where M denotes the metal ion, n is the solvation number, and z represents the oxidation state of the cation. These metal–ion complexes function as Lewis acids, promoting sugar isomerization and subsequent dehydration–condensation reactions [14,37]. As illustrated in Figure 8, a possible reaction mechanism for bamboo conversion into HMF involves a two-step treatment consisting of ultrasonic–formic acid pretreatment followed by a ChCl–citric acid/CaCl_2_ catalytic system.

During the ultrasonic–formic acid pretreatment, the cavitation effect produces localized high-temperature and high-pressure microenvironments that disrupt the chemical bonds linking lignin with cellulose and hemicellulose, thereby facilitating efficient lignin removal. This process simultaneously increases the surface area and accessibility of cellulose, enhancing its overall chemical reactivity. In addition, ultrasound treatment increases cellulose crystallinity, which further improves its enzymatic hydrolysis efficiency. In the subsequent catalytic stage, citric acid acts as a hydrogen-bond donor (HBD) and choline chloride as a hydrogen-bond acceptor (HBA), together forming a deep eutectic solvent (DES). The resulting hydrogen-bonding network stabilizes the DES structure and provides an acidic microenvironment conducive to the dehydration of glucose to HMF.

The conversion of glucose to HMF involves two key steps: isomerization of glucose to fructose, followed by dehydration of fructose to HMF. Glucose directly coordinates with the Lewis acidic sites of Ca^2+^, stabilizing its ring-opened form and thereby promoting isomerization to fructose. In parallel, Brønsted acidic sites provided by citric acid protonate hydroxyl groups, generating intermediates that are more susceptible to dehydration. For instance, during fructose dehydration to form HMF, protonation of hydroxyl groups facilitates the removal of water molecules and accelerates HMF formation. Furthermore, Ca–substrate complexes coordinate with oxycarbonyl groups, promoting the formation of enol intermediates, which subsequently undergo tautomerization to yield aldehyde species, serving as key intermediates in the HMF synthesis pathway [40].

Subsequently, the acidic system promotes the dehydration of intermediates, leading to the formation of HMF. However, when the reaction was carried out in the absence of DES, black solid residues were observed in the reaction mixture. This phenomenon may be attributed to the gradual condensation of HMF molecules into a humin structure—a furan-crosslinked polymer formed by acetalation, etherification, esterification and aldol condensation [41,42,43]. Its high density of accessible hydroxyl functionality has recently been shown to improve the performance of MOF materials [44]. In addition, alkaline conditions tend to generate structures enriched in aromatic hydrocarbons and carboxylic acids, whereas acidic conditions are more favorable for humin formation. High temperatures and prolonged reaction times further accelerate humin generation [43]. The incorporation of ChCl promotes the formation of a hydrogen-bonding network that stabilizes reactive intermediates, thereby reducing humin production [45,46]. Furthermore, ChCl acts synergistically with acidic catalysts by modulating the reaction acidity and suppressing over-condensation of intermediates, effectively inhibiting humin formation [47].

## 4. Conclusions

In this study, 83.7% hemicellulose and 76.5% lignin were removed from raw BP following ultrasonic-assisted formic acid pretreatment. Meanwhile, an acidic DES system was generated from ChCl and citric acid—an intrinsic buffer component of the enzymatic hydrolysate—thereby eliminating the need for an external Brønsted acid. The incorporation of Ca^2+^ as a synergistic Lewis acid catalyst further enabled efficient HMF production. When 100 mM Ca^2+^ was combined with a 2:1 ChCl–citric acid ratio, an HMF yield of 51.9% was achieved. Based on these findings, a plausible reaction mechanism is proposed for glucose conversion to HMF within the ChCl–citric acid/Ca^2+^ system: citric acid facilitates fructose dehydration to generate reactive intermediates, while Ca^2+^ and ChCl promote enol formation through coordination interactions. These intermediates then undergo tautomerization to form aldehyde species, which subsequently dehydrate under acidic conditions to yield HMF.

## Figures and Tables

**Table 1:** Previously reported HMF yield from different biomasses and conditions.

Biomass	Catalysts and Conditions	Yield	References
Bamboo	microwave-H_2_SO_4_, 140 °C 5 min	10.94%	[9]
Poppy straw	CuCl_2_, 200 °C, 30 min	12.24%	[9]
Rice straw	NaOH pretreatment, HSO_3_-ZSM-5 zeolite, 160 °C, 5 h	54.1%	[11]
Bamboo culm	microwave, 170 °C, 0.13 M HCl	37%	[12]
Bamboo	Ultrasonic-Formic Acid Pretreatment CaCl_2_, DES, 220 °C, 1.5 h	51.9%	This work

**Table 2:** Peak assignments for FT-IR spectra.

Wavenumbers/cm^−1^	Assignments
3350	O-H stretch vibration
2900	C-H stretch in methyl groups
1730	C=O stretch vibration
1600, 1510, 1422	Aromatic skeletal vibrations
1240	the ether bond
1050	C-O expansion vibration of cellulose

**Table 3:** XPS analysis of untreated and differently pretreated solid samples.

Sample	O%	C%	O/C	C1%	C2%	C3%
Raw BP	26.73	73.27	0.365	46.95	47.54	5.52
US-BP	28.52	71.48	0.399	43.56	50.37	6.07
S-FA-BP	33.79	66.21	0.51	30.17	65.77	4.06
US-FA-BP	35.97	64.03	0.562	20.52	75.48	4.00

## Data Availability

The original contributions presented in this study are included in the article. Further inquiries can be directed to the corresponding author.
